# The Influence of Fermentation Technology on the Functional and Sensory Properties of Hemp Bread

**DOI:** 10.3390/molecules29225455

**Published:** 2024-11-19

**Authors:** Stanisław Kowalski, Anna Mikulec, Dorota Litwinek, Barbara Mickowska, Magdalena Skotnicka, Joanna Oracz, Kaja Karwowska, Anna Wywrocka-Gurgul, Renata Sabat, Anna Platta

**Affiliations:** 1 Faculty of Food Technology, Department of Carbohydrate Technology and Cereal Processing, University of Agriculture in Krakow, 30-149 Krakow, Poland; dorota.litwinek@urk.edu.pl (D.L.); anna.wywrocka-gurgul@urk.edu.pl (A.W.-G.); renata.sabat@urk.edu.pl (R.S.); 2 Faculty of Engineering Sciences, University of Applied Science in Nowy Sacz, 33-300 Nowy Sacz, Poland; amikulec@ans-ns.edu.pl; 3 Department of Plant Product Technology and Nutrition Hygiene, Faculty of Food Technology, University of Agriculture in Krakow, 30-149 Krakow, Poland; barbara.mickowska@urk.edu.pl; 4 Department of Commodity Science, Faculty of Health Sciences with the Institute of Maritime and Tropical Medicine, Medical University of Gdansk, 80-210 Gdansk, Poland; skotnicka@gumed.edu.pl (M.S.); kaja.karwowska@gumed.edu.pl (K.K.); 5 Institute of Food Technology and Analysis, Faculty of Biotechnology and Food Sciences, Lodz University of Technology, 90-924 Łodz, Poland; joanna.oracz@p.lodz.pl; 6 Faculty of Management and Quality Science, Gdynia Maritime University, 81-225 Gdynia, Poland; a.platta@wznj.umg.edu.pl

**Keywords:** hemp flour fermentation, flavor and aroma profile, correlation of sensory and instrumental analysis, nutritional properties of hemp bread, amino acid composition, fatty acid profile

## Abstract

In this work, the type of fermentation and baking technology used to make hemp bread was investigated. The physicochemical composition of flour and bread, the protein nutritional quality, fatty acids profile, texture, consumer acceptance, analysis of volatile compounds using an electronic nose and chemical compounds using an electronic tongue were determined. Differences in protein and total dietary fiber content were observed in the obtained breads. The use of sourdoughs had a minor effect on the physical properties of the bread tested (the volume and mass of the loaves, color, or crumb texture). There was no clear effect of the tested sourdoughs on the parameters of the crumb texture and its moisture, assessed physically, both on the day of baking and during storage. In this bread, the limiting amino acid was lysine (amino acid score from 56.22% to 57.63%), but the breads did not differ significantly in the value of this indicator. The n-6/n-3 ratio in breads containing hemp flour (from 3.73 to 4.48) may have a beneficial effect on human health. The best-rated bread was the HB4 with a score of 6.09. The acceptance of remaining breads were in the range from 3.91 for HB1 to 4.91 for HB2.

## 1. Introduction

Hemp (*Cannabis sativa* L.) is a high-yielding, annual, industrial plant cultivated for its stem fibers and seed oil. For years it was considered a niche plant, but currently its cultivation and production is experiencing a renaissance. It is cultivated in over 30 countries, and China is the largest producer and exporter of hemp [1].

The cultivation and production of hemp is in line with the policy announced by the Food and Agriculture Organization of the United Nations (FAO) and the World Health Organization (WHO) in 2019. A new definition and guiding principles for sustainable and healthy nutrition have been published, which are based on a holistic approach to diet, paying particular attention to the three pillars of sustainable development: environmental, social/cultural and economic sustainability [2].

The term “hemp” refers to varieties of *Cannabis sativa* grown for industrial purposes containing delta-9-tetrahydrocannabinol (THC) in trace amounts. Commission Regulation (EU) 2022/1393 sets maximum levels of delta-9-tetrahydrocannabinol (Δ9-THC) in hemp seeds and products derived from hemp seeds as delta-9-tetrahydrocannabinol (Δ9-THC) equivalents. The maximum level relates to the sum of delta-9-tetrahydrocannabinol (Δ9-THC) and delta-9-tetrahydrocannabinolic acid (Δ9-THCA), expressed as Δ9-THC. The maximum level as delta-9-tetrahydrocannabinol (Δ9-THC) equivalents is 3 mg/kg for hemp seed, ground hemp seed, (partially) defatted hemp seed and other products processed/derived from hemp seed and 7 mg/kg for oil from hemp seeds. In recent years, there has been an increase in the number of non-pharmacological cannabis varieties with low delta-9-tetrahydrocannabinol (Δ9-THC) content [3].

Fortified products are often more preferred because consumer needs and demands have changed significantly in recent years. Modern consumers no longer treat bread solely as a source of energy, but expect it to be a product of high nutritional value, enabling the implementation of a nutritious and functional diet [4]. Due to their valuable chemical composition and nutritional value, hemp seeds seem to be a good choice for fortifying bakery products.

Hemp seeds are a valuable source of protein (about 25%), unsaturated fats, dietary fiber, vitamins and minerals [5]. The dominant proteins in hemp seeds are mainly albumin and edestin, which have a significant content of exogenous amino acids [6] and are additionally classified as non-allergenic [7]. The amino acid profile of hemp seeds is similar to that of chicken eggs and soybeans [8]. It is worth emphasizing that hemp products are gluten-free, so they can be successfully used in the nutrition of people suffering from food intolerances, such as celiac disease, replacing cereal products (wheat, barley, or rye) containing gluten.

Of all fats, 70–80% are polyunsaturated fatty acids, which are a valuable source of essential omega-3 fatty acids. Additionally, hemp oil has one of the lowest n-6/n-3 ratios found in nature [9], which is considered optimal for human health [10]. Furthermore, hemp seeds have favorable contents of phenolic compounds, phytosterols, and tocopherols [11,12], which are key features of functional foods, as well as a high antioxidant capacity [12,13]. They are also a valuable source of minerals such as iron, calcium, magnesium, and manganese [9]. It is worth emphasizing that the concentration of antinutritional compounds in hemp seeds is very low [14], which makes them an interesting alternative to concentrates and meals, e.g., from legumes. Antinutritional compounds in hemp seed include phytic acid, condensed tannins, cyanogenic glycosides, and trypsin inhibitors [14]. Phytic acid reduces the digestibility of proteins and increases the excretion of endogenous nitrogen, amino acids, and minerals, particularly divalent cations [15]. Condensed tannins (flavan-3-ol-based biopolymers) negatively affect the absorption of nitrogenous compounds and minerals [16]. Cyanogenic glycosides (saponins), due to their irritating taste, may contribute to lower intake of foods containing cannabis seeds and also slow intestinal peristalsis, intestinal motility and, due to the formation of difficult-to-digest saponin–protein complexes, reduce protein digestibility [17]. Trypsin inhibitors are considered one of the most important antinutritional agents and are found in many graminaceous, cruciferous, and leguminosae plant species. Their antinutritional effect is through inhibition of the activity of enzymes that break down peptide bonds, which reduces protein digestibility [18].

The first breads were fermented exclusively with the participation of natural flour microflora. Currently, there is a return to ancient techniques of bread production with the use of sourdough. The use of sourdough affects a number of quality features of bread, generally improving the taste and aroma of bread, delaying the staling process, or improving texture features [19]. Long fermentation also affects the nutritional value of bread. The course of the fermentation process and the metabolites formed depend on many factors, such as fermentation conditions, the type of dominant microflora, the composition and size of flour particles, protein content and its quality, starch properties, and dietary fiber or enzymatic activity of flours [19,20].

The aim of the work was to investigate the influence of the type of fermentation and baking technology of hemp bread.

## 2. Results and Discussion

### 2.1. Basic Physicochemical Characteristics

No significant differences were observed in the content of protein, fat, ash, and insoluble and soluble fractions of dietary fiber, while statistically significant differences in total dietary fiber (between HB3 and HB1) will not affect the nutritional value of the product (Table 1). The fermentation carried out was probably too short to significantly modify the chemical composition of the obtained breads. The obtained results are consistent with those already described in the literature [12,21].

The same fermentation conditions (time, temperature, type of sourdough) and the same starter cultures (dedicated to each type of flour) were used in the studies, modifying the sourdough by using different flours. In this way, three sourdoughs were obtained: wheat (SW), wheat-hemp (SHW) and hemp (SH) with different quality features. Significant differences were observed in the rate of souring of the individual sourdoughs. It is worth noting that the pH of hemp flour was lower than that of wheat flour, hence the lower initial acidity of wheat flour. Hemp flour in SHW sourdough constituted 50%, which did not significantly lower the pH of the mixture itself, and the buffering properties of the flour allowed for maintaining a higher pH value [22]. The sourdoughs made of wheat flour and water alone soured the fastest; a clear difference between the sourdoughs was already visible after 1.5 h of souring, when the sourdough made only from wheat flour had an acidity of 5.56, while the other sourdoughs had an acidity of approximately 5.91 (Figure S1). The wheat sourdough soured more dynamically after 11 h of fermentation and reached pH 4 after 16 h, the other sourdoughs soured more strongly only after 14 h of fermentation and reached a much higher pH. The wheat-hemp sourdough after 24 h of fermentation was characterized by a pH of 4.12, while the hemp sourdough only 5.41, which indicates that the pH of the sourdough was too high. Usually after 10 h of fermentation the sourdoughs should have achieve a pH in the range of 3.3–4.3 [20].

The reason for the lower degree of acidification of these sourdoughs may be too few fermentable sugars in hemp flour as well as too much dietary fiber, which probably contributed to the significant water binding by this fraction and could limit the fermentation process, but there is a lack of clear research in this area [19]. The higher protein content in hemp flour is also significant. Although, as shown by Arend et al. [23], sourdoughs made from whole grain high-fiber wheat flour contained significantly more lactic and acetic acid compared to sourdoughs from low-fiber flour, it should be borne in mind that this is a different type of protein. At the same time, it should be remembered that a lower pH also affects proteins, increasing their solubility, especially at pH below 4, which consequently limits the formation of bonds and weakens the gluten network, influencing the rheology of the dough [23].

The use of sourdoughs made from different flours had a minor effect on the physical properties of the bread tested, such as the volume and mass of the loaves, color, or crumb texture. The use of sourdoughs had a negative effect on the volume of the loaves, as bread made only from wheat and hemp flour without sourdough (HB1) had a significantly larger volume compared to breads with sourdoughs (HB2, HB3, and HB4). The volume of the breads was directly proportional to the remaining volume parameters, i.e., specific volume or volume-yield (Table 2).

Literature information on the effect of lactic fermentation of wheat flour on the volume of bread is varied. On the one hand, it is said that a lower volume of bread could be obtained, which results from the weakening of gluten structures as a result of lowering the pH of the environment, especially gliadin, which is mainly responsible for the volume and texture of the product [24]. At the same time, the important role of exopolysaccharides (EPS) produced during sourdough fermentation by lactic acid bacteria in creating the structure is emphasized, as it has been shown to have a beneficial effect on a number of technological properties of bread, including water absorption by the dough, increased loaf volume, and delayed bread staling [25].

Long fermentation may also contribute to the lightening of products due to the oxidation of colored compounds under the influence of acids formed during the fermentation process [26]. In the studies of this work, no clear effect on the color of the crumb was observed. It was found that wheat sourdough breads had a significantly darker color compared to the other breads, but a greater saturation of yellow and red color was observed after the use of all sourdoughs. Based on delta E, it was found that a slight difference in color between the samples should be visible to the consumer (Table 3).

There was no clear effect of the use of the tested sourdoughs on the parameters of the crumb texture and its moisture, assessed physically, both on the day of baking and during storage. The storage time had a much greater effect on the crumb texture because with storage, the hardness and chewiness of the crumb increased, and its cohesion and resilience decreased, which are typical changes occurring during bread storage [27]. The main factor influencing the improvement of the textural properties of bread crumb is the increase in the amount of soluble dietary fiber in the bread [24], which was not observed in the tested breads (Table 4).

### 2.2. Amino Acid Composition

Proteins are among the most important nutrients that are essential for maintaining life, and their adequate supply determines proper growth and the repair and creation of new tissues [28]. Nowadays, it is common to use different protein sources to improve the biological value of food products [29,30]. Although animal proteins are usually characterized by a full amino acid profile, compared to plant proteins, plant proteins are very popular with consumers due to the popularity of a vegan lifestyle, sustainable development, and ethical issues, which means that the food industry, in response to customers’ demands, is creating an increasingly large range of such products [31]. Hemp (*Cannabis sativa* L.) is known for its high digestibility and favorable amino acid composition [32]. Moreover, hemp proteins contain significantly higher amounts of free sulfhydryl groups than soy protein [33]. The sum of exogenous amino acids in the raw materials was significantly higher in hemp flour compared to wheat flour. However, in the obtained breads, only hemp bread with hemp sourdough was characterized by a significantly lower content of exogenous amino acids (Table 5). Comparing the content of individual exogenous amino acids in the breads, it can be stated that hemp bread with hemp sourdough contained significantly less leucine, threonine, and valine compared to the other samples (Table 4).

Raw materials and products containing wheat flour were characterized by a significantly higher total content of endogenous amino acids (Table 5). The limiting amino acids in wheat flour were valine and lysine, whereas in the case of hemp flour, they were leucine and lysine. In the obtained breads, the limiting amino acid was lysine, but the breads did not differ significantly in the value of this indicator (Table 6).

Enzymatic hydrolysis or lactic acid fermentation is commonly used to increase the functional and biological value of some proteins [34]. Lactic acid fermentation causes biochemical changes in components, including proteins [35]. Bartkiene et al. [36] studied the effect of *P. acidilactici* LUHS29 and *P. pentosaceus* LUHS18 strains on the fermentation of various proteins, including hemp protein powder. They observed that proteins fermented with *P. acidilactici* had a noticeably higher concentration of both exogenous and endogenous amino acids than those fermented with *P. pentosaceus*. The AAS index for lysine obtained in the obtained breads is over 10% higher than the AAS value for lysine in wheat bread, which usually does not exceed 50% [37]. This is particularly important because the human body is unable to produce this amino acid; therefore, it should be obtained from food.

### 2.3. SDS-PAGE Electrophoresis

The majority of cereal grains proteins are storage proteins (70–90%) with biological functions to supply the seedling with nitrogen and amino acids during germination. The remaining minority, albumins and globulins, are metabolic proteins (e.g., enzymes or enzyme inhibitors) [38,39].

Wheat storage proteins are complexed to gluten, consisting of two fractions: prolamins and glutelins (with a ratio of about 1:1). The molecular weight of native gluten proteins ranges from around 30,000 to more than 10 million Da, but under denaturing and reducing conditions of SDS-PAGE wheat prolamins (gliadins) are visible as protein bands with molecular weights between 28 and 55 kDa, and glutelins 32 and 88 kDa [40]. In our electrophoresis experiment, prolamin proteins represent the dominating fraction of wheat flour and are visible in the expected range of molecular weight. A very similar protein profile is also observed for wheat sourdough, although there is a noticeable decrease in the amount of these prolamin proteins due to limited proteolysis during sourdough fermentation (Figure S2, lanes 5–6).

In the case of hemp flour, the proteins consist mainly of globulin edestin (60–80% of total protein) and albumin [41,42]. Edestin subunits are visible in the molecular weight range 18–23 kDa and approx. 35 kDa, and an additional albumin band of about 10 kDa. There is no difference in protein profiles of hemp flour and hemp sourdough, in contrast to mixed sourdough, where there is a lack of protein bands in the range of 15–23 kDa, and a noticeable increase in small proteins in the range of 10–13 kDa, which must have been caused by proteolytic activity of proteinases during fermentation (Figure S2, lanes 7–9). The protein patterns of all examined breads are similar, with no significant differences, but both cereal species components are clearly visible—major wheat and hemp proteins. Interestingly, in the case of hemp proteins, only bands visible in hemp sourdough remained in bread, which means that proteolytic activity present in wheat flour also hydrolyzed proteins of hemp flour (just as in mixed sourdough) (Figure S2, lanes 1–4).

### 2.4. Fatty Acid Profile

Omega-6 and omega-3 polyunsaturated fatty acids are essential fatty acids that must be supplied in the diet. It is usually indicated that due to the lack of appropriate enzymes (desaturases introducing double bonds at carbon 3 and 6 in the carbon chain), the human organism does not synthesize linoleic acid (LA) and α-linolenic acid (ALA), and therefore, they constitute a pool of so-called essential unsaturated fatty acids. They are precursors for the synthesis of long-chain polyenoic fatty acids such as eicosapentaenoic acid (EPA) and docosahexaenoic acid (DHA) [43]. In the fatty acids profile, the most abundant was conjugated linoleic acid—CLA (C18:2 cis), which was most abundant in hemp flour (55.33%). Its amount differed significantly in individual breads. The highest amount was found in hemp bread with SHW sourdough (49.33%) and the lowest in hemp bread without sourdough (40.29%). (Table 7). The second most abundant was α-linolenic acid (ALA) (18:3n3), which was significantly more abundant in hemp flour (17.17%) compared to wheat flour (4.16%). The breads also differed significantly in its content. The hemp bread with hemp sourdough contained the most ALA (11.53%), compared to the hemp bread with the least (10.54%). A high content of polyunsaturated fatty acids (PUFA), 77.60%, was characteristic of hemp flour, while in hemp bread, it ranged from 54.46 for HB1 to 64.55% for HB3 (Table 7).

The dominant, relatively low PUFA/SFA ratio in the diet is associated with a risk factor for elevated blood cholesterol levels. In this study, the use of hemp meal allowed us to obtain a satisfactory PUFA/SFA ratio, which ranged from 4.07 for bread with wheat-hemp sourdough to 4.90 for hemp bread with hemp sourdough. A high level of α-linolenic acid led to a significant reduction in the n-6/n-3 ratio in the obtained breads, while for wheat bread, this quotient in the study by Kowalski et al. [45] was as much as 18.77. The optimal n-6:n-3 ratio should be between 1:1 and 5:1 to maintain a healthy balance in the body. ALA and linoleic acid (LA) are metabolized in competing common enzymatic reactions; therefore, increasing n-6 PUFA intake through LA consumption may inhibit the synthesis of eicosapentaenoic acid (EPA) and docosahexaenoic acid (DHA) from n-3 PUFA. This is important because the typical Western diet is characterized by a high intake of n-6, which increases the n-6:n-3 ratio in the range from 10:1 to 20:1, which increases the risk of developing inflammatory diseases and obesity [46,47]. Therefore, the n-6/n-3 ratio in hemp flour and breads containing it, ranging from 3.38 for hemp to 4.48 for hemp bread with wheat-hemp sourdough, may have a beneficial effect on human health.

### 2.5. Consumer Acceptance

**Figure 1:**
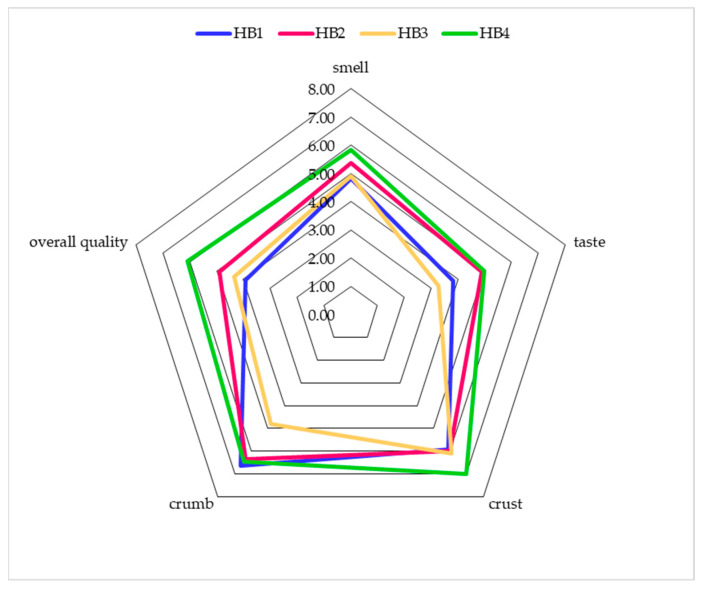
Acceptance assessment of bread with the addition/participation of hemp flour (HB1—hemp bread; HB2—hemp bread with wheat sourdough; HB3—hemp bread with wheat and hemp sourdough; HB4—hemp bread with hemp sourdough).

Consumer preference assessments constitute important and valuable information for producers, and in a market economy, they constitute one of the basic factors in the decision-making process regarding the purchase of a food product, determined, among others, by the brand, organoleptic characteristics, type and size of packaging and price. In terms of consumer acceptance, the breads did not differ significantly for most parameters, although hemp bread with the addition of hemp sourdough received the highest rating. Only significant differences were observed in the assessment of acceptance of the crumb and the overall quality of the breads. The HB3 bread for crumb acceptance obtained a lower average score than the other breads, which was 4.82, and the other breads obtained scores from 6.36 for HB2 and 6.64 for HB1; these breads did not differ significantly from each other (Figure 1). However, in the acceptance of the overall quality, the HB4 bread obtained a significantly higher average than the others, which was 6.09. The remaining breads did not differ from each other, obtaining from 3.91 for HB1 to 4.91 for HB2 (Figure 1).

The obtained results are consistent with previous studies, in which breads with 15, 30, and 50% of hemp flour were obtained. In the organoleptic assessment, lower scores were observed for individual evaluated parameters, such as aroma, taste, texture, crumb and crust color, and overall acceptability. The average number of points decreased with the increase in the share of hemp flour [12]. Also, Švec et al. [48] observed lower organoleptic assessment and acceptance of bread by consumers, where the assessors additionally indicated an unfavorable hay-like and bitter aftertaste of bread with hemp flour. Capacanari et al., who added up to 40% hemp seed cake flour to bread, also observed that the consumer assessment decreased with the increase in the content of hemp seed cake flour in breads [49]. It is worth noting that the use of the fermentation process has had an impact on reducing the earthy aroma and the bitter and sour taste. In turn, the flavor profile has become dominated by cereal and malt flavors.

### 2.6. Quantitative Descriptive Analysis

A manufacturer must know not only how a product is rated by consumers but also why it received a specific rating. The QDA sensory profiling method assumes that sensory attributes are not homogeneous attributes but consist of a number of individual attributes, a significant portion of which can be identified and analyzed separately. In the aroma profile, earthy and maritime dominated in HB1 and HB3 breads, mill in HB2, and mill, earthy and nutty in HB4 (Figure S3). In the taste profile, bitterness dominated in all breads, although with different intensities, from 4.11 for HB4 to 7.55 for HB1. Additionally, sourness also dominated in HB1, cereal, and word in HB2 and HB3, and malt in HB4 (Figure S3). In the crust profile, brownness, hardness, and crispiness were most noticeable. The highest scores were given in HB1 bread, with responses of 8.01, 7.90, and 7.74. In the crumb profile, brownness dominated in all breads, greyness in HB1-HB3 breads, and porosity in HB3 and HB4 (Figure S3).

### 2.7. Clustering of Sensory and Instrumental Data

The clustering method is particularly effective for identifying natural groupings or patterns within complex, high-dimensional datasets. Clustering serves to reduce the complexity of the dataset by organizing it into manageable groups, allowing for clearer interpretation and facilitating meaningful comparisons between different bread types. It also enables the integration of multiple data sources, in this case, subjective human sensory evaluations and instrumental analysis (e-nose and e-tongue measurements) (Tables S1 and S2). If both data sources align, bread types with similar characteristics will cluster together, providing an opportunity to validate the consistency between human assessments and device readings.

The analysis of clusters provides insights into the distinguishing characteristics of each group, highlighting specific features that define them. If a particular cluster exhibits a significantly higher average for a given feature, it indicates that the cluster is primarily characterized by that attribute (Table S3).

Cluster 0 shows a notably higher average value for the GPS feature (8.6) compared to an average of around 5.3 in the other clusters. A pronounced difference is also observed in the human taste attribute, where the value for cereal is the highest among the three clusters (6.5), indicating that bread samples in this cluster are strongly associated with this smell profile. This cluster includes bread HB2 and HB3 share similar sensory and analytical profiles.

Cluster 1 was distinguished by its device smell attributes, particularly for ethanol, where the average value is approximately 77, compared to 67 in Clusters 0 and 2. In contrast, Cluster 1 has lower average values for all other device smell features when compared to the other clusters, suggesting a unique smell profile. This cluster includes bread HB4 stands out with distinct attributes, forming its own cluster.

Cluster 2 stands out with a significantly lower average value for SRS (3.5), while the corresponding value in other clusters is around 7.5. Additionally, the average for SPS is higher in this cluster (8.2) compared to around 6.5 in the others. In terms of human sensory data, Cluster 2 has the highest average scores for the earthy and maritime smell attributes (7). Regarding taste, Cluster 2 has the highest average score for sour (6.6) and the lowest score for malty, at 2, which sets it apart from the other clusters. This cluster included bread HB1 is grouped separately, indicating unique characteristics compared to the others.

The silhouette score of 0.6628 indicates a favorable clustering outcome. This score suggests that the clusters are well-separated, with most data points accurately assigned to their respective clusters.

The original dataset’s high dimensionality posed challenges for direct visualization. To address this, Principal Component Analysis (PCA) was used to reduce the data to two principal components, which serve as a compressed representation of the original features. In the resulting plot, the x-axis and y-axis correspond to Principal Component 1 (PC1) and Principal Component 2 (PC2).

**Figure 2:**
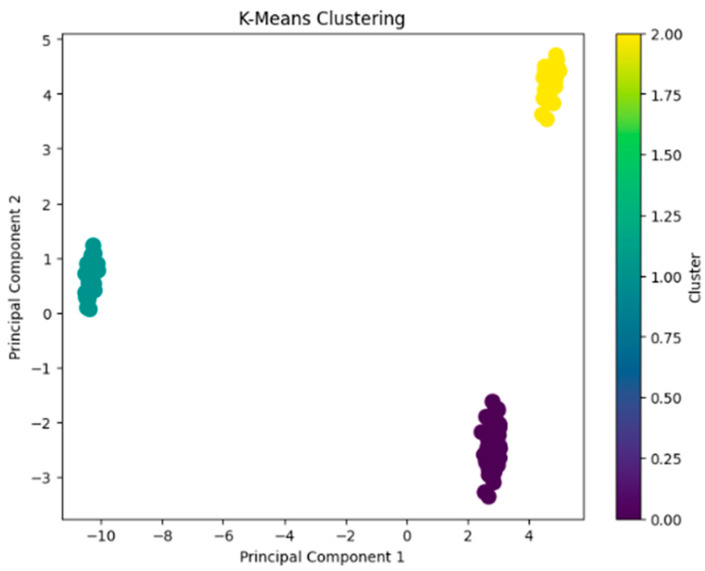
PCA analysis graph showing the grouping of individual breads.

The plot reveals three distinct groups (Figure 2). This indicates that the clustering process has effectively identified different patterns within the dataset. The degree of separation between the clusters confirms their distinctiveness, highlighting that the features related to taste and smell attributes result in well-defined groups.

Hemp as a food product has pleasant and unique sensory characteristics, such as taste, mainly due to its terpenoid content. Some volatile compounds, such as α-humulene, caryophyllene, α- and β-pinene, myrcene, or terpinolene from hemp inflorescences, can also be used as flavoring agents in food production [32,50]. Due to the high content of volatile compounds, hemp products have been studied by other authors for their effect on the sensory characteristics of food products. For example, Lukin and Bitiutskikh [51] observed a pleasant nutty flavor in breads with hemp flour. Merlino et al. [52] observed that adding hemp flour to gnocchi improved the sensory attributes to some extent, but the bitter taste of hemp negatively affected the final product, reducing its overall acceptability.

## 3. Materials and Methods

### 3.1. Preparation of the Sourdough

Sourdough loaves were prepared using a two-phase method; the first phase of sourdough was prepared according to the following procedure: 70 g of flour (100% wheat flour or 50% hemp flour and 50% wheat flour or 100% hemp flour) and 1.4 g of commercial starter cultures—LV1 (SAF LEVAIN; Lesaffre, Wolczyn, Poland) were mixed together with 140 mL of water. LV1 SAF Levain containing a mixture of *L. casei* and *L. brevis* cultures (2% in total) with *S. chevalieri* yeast (98%) [53]. Afterward, the sourdoughs were fermented at 30 °C for 6 h in a laboratory incubator (IPS, Memmert GmbH & Co. KG, Schwabach, Germany). The second phase of sourdough was prepared by adding 210 g flour to the first phase (100% wheat flour or 50% wheat flour and 50% hemp flour or 100% hemp flour) and 420 mL of water and fermented for the next 18 h at 30 °C. The obtained leavens were marked as SW (100% wheat flour), SH (100% hemp flour), SHW (50% wheat flour and 50% hemp flour), respectively. The pH of sourdoughs was measured during fermentation using a digital high-performance meter ProLab 2500 (SI Analytics GmbH, Mainz am Rhein, Germany), equipped with an electrode BlueLine 14 pH (SI Analytics, Germany).

### 3.2. Bread Production

Three types of bread were prepared with 15% of hemp flour addition, the breads differed in the type of sourdough added. The reference sample was hemp bread without any sourdough starter, only made with yeast.

#### 3.2.1. Standard Bread

In total, 850 g of wheat flour, 150 g of hemp flour, 670 mL of water, 18 g of salt, and 10 g of baker’s yeast were mixed for 12 min in the spiral mixer (type SP12, Diosna Dierks & Söhne GmbH, Osnabrück, Germany). After the end of mixing, the dough was left at 40 °C for 30 min. Next, 250 g of dough pieces were then formed and finally fermented for 30 min in a proofer oven at a temperature of 40 °C and 90% RH (MIWE Condo type CO 2.0608 electric oven, MIWE GmbH, Arnstein, Germany). The bread was baked at 210 °C for 40 min in an electrically heated deck oven MIWE Condo (MIWE Michael Wenz GmbH, Meiningen, Germany). This bread is marked with the abbreviation HB1.

#### 3.2.2. Sourdough Breads

A total of 450 g of sourdough (from 100% wheat flour (SW) or 50% hemp flour and 50% wheat flour (SHW) or 100% hemp flour (SH)) were mixed for 12 min with the remaining flour (weight of flour in the final dough 1000 g), 370 mL of water, 18 g of salt and 10 g of baker’s yeast was mixed for 12 min in the spiral mixer (type SP12, Diosna Dierks & Söhne GmbH, Osnabrück, Germany). The dough was fermented and baked in the same way as standard bread. The obtained breads were marked as HB2, HB3, HB4, respectively.

### 3.3. Analysis of Basic Features

The following parameters characterizing the quality of bread were determined in the finished product: total baking loss [54]; bread volume was established by means of a three-dimensional analysis using a low-frequency, high-precision laser Volscan Profiler (Stable Microsystems, Godalming, UK). Based on the bread size in the study, a vertical step size of 2 mm and a rotational speed of 0.5 rps were applied, and crumb moisture was estimated using the gravimetric method no. 925.10 [55].

The chemical parameters of wheat and hemp flour were also subjected to analysis: ash content (AOAC 923.03); protein content as the sum of amino acids according to the methodology in Section 3.5; crude fat content (AOAC 935.38); water content (AOAC 925.10); total, soluble, and insoluble dietary fiber content (AOAC 991.43) [55]. Available carbohydrate contents were calculated according to FAO/WHO [56]. Analyses were performed in triplicate.

### 3.4. Color Analysis

Color analysis was performed according to the CIElab (L*, a*, b*) system using a Konica Minolta CM-5 spectrophotometer (Konica Minolta Sensing, Osaka, Japan) [12].

### 3.5. Amino Acid Composition

Amino acids were determined by ion-exchange chromatography with strong cation ion-exchanger and sodium-citrate elution buffers system followed by post-column derivatization with ninhydrin and spectrophotometric detection at 570 and 440 nm, according to the standard protocol of manufacturer of amino acid analyzer [Ingos, Prague, Czech Republic] preceded by acid hydrolysis of proteins in liquid phase based on the work of Moore and Stein [57]; Davidson [58] and Smith [59]. Sulfur-containing amino acids were analyzed as oxidation products obtained by performic acid oxidation followed by standard hydrolysis procedure. For calibration of the amino acid analyzer, the amino acid standard solution was used (Sigma, St. Louis, MO, USA). Evaluation of the acquired data was performed using the software of a chromatographic device (Chromulan, Pikron, Prague, Czech Republic).

### 3.6. The Protein Nutritional Quality

Nutritional value of protein expressed as Amino Acid Score (AAS) was calculated [2] (Equation (1)):

AAS = (mg of amino acid in 1 g of test protein)/(mg of amino acid in reference pattern *),(1)

* recommended amino acids scoring patterns for adolescents and adults [60].

### 3.7. SDS-PAGE Electrophoresis

Lyophilized samples were dissolved and incubated in Complete Protease Inhibitor Cocktail (Roche, Basel, Switzerland), followed by extraction directly in denaturing and reducing sample buffer (125 mM Tris-HCl pH 6.8, 4% SDS, 20% *v*/*v* glycerol, DTT 50 mg/mL). After centrifugation, samples were boiled at 100 °C for 5 min. SDS-PAGE electrophoresis was performed on Any-kD™ Mini-PROTEAN^®^ TGX™ Precast Protein Gels (Bio-Rad, Hercules, CA, USA) with Tris-Glycine-SDS Laemmli running buffer and a voltage of 200 V. PageRuler Prestained Protein Ladder, molecular weight range 10–170 kDa, was used as molecular weight protein markers (Thermo Fisher Scientific, Waltham, MA, USA). Gels were run in Mini Protean Tetra Cell electrophoresis equipment (Bio-Rad, Boulder, CO, USA) and stained with Coomassie Brillant Blue R-250.

### 3.8. Determination of Fatty Acid Profile

The extraction of total lipids was performed in triplicate by gas chromatography method according to AOAC method 935.38, while the derivatization and determination of total fatty acids composition were performed according to the AOAC-approved method 991.39 [55]. Shimadzu GC2010Plus Chromatograph (Shimadzu corp., Kyoto, Japan) with flame ionization detector (FID) was used to determine the fatty acid profile. The operating parameters were as follows: FID detector temperature 240 °C; temperature dispenser 240 °C; oven temperature 195 °C to 240 °C (5 °C/min) (240 °C, 10 min). SH-FAME column (30 m–0.32 mm–0.25 μm) was used, and carrier gas helium 1.6 cm^3^/min., split ratio 100. Individual fatty acid methyl esters were identified by comparison to the standard mixture of Supelco 37 component FAME Mix, Sigma-Aldrich Co., and of CLA isomers (Sigma-Aldrich Co., St. Louis, MO, USA). Analyses was performed in three repetitions.

### 3.9. Analysis of Volatile Compounds Using the Electronic Nose

The method described by Żyżelewicz et al. [61], was used for the analysis of volatile flavor compounds. The E-nose analysis was performed using a Heracles II electronic nose (Alpha MOS, Toulouse, France). The retention times of the n-alkanes were employed to determine the Kovats indices and identify the volatile compounds utilizing AromaChemBase software (Alpha MOS, Toulouse, France). Each sample was measured in triplicate. Instrument control, data acquisition, and evaluation were conducted using Alphasoft 14.2 and AroChembase software (Alpha MOS, Toulouse, France).

### 3.10. Analysis of Chemical Compounds Using Electronic Tongue

The Alpha MOS ASTREE II electronic tongue (e-tongue) instrument (Alpha MOS, Toulouse, France) was used for instrumental taste analysis of the bread samples. The sensor set #5, designed for food and beverage applications, was employed [62]. This sensor (set #5) consisted of seven sensors (SRS, GPS, STS, SPS, UMS, SWS, and BRS) and a reference electrode (Ag/AgCl). The analysis was performed five times. AlphaSoft software (Alpha MOS, Toulouse, France) was used for instrument control, data acquisition, and processing. Taste screening analysis was used to rank the samples based on taste attributes using a scale of 0 to 12 for intensity.

### 3.11. Texture Analysis

Texture parameters were measured using a texture analyzer TA.XT Plus (Stable Microsystems, Godalming, UK) according to the standard program, at the compression rate 5 mm/s. A 10 mm diameter cylinder of bread crumb with a height of 15 mm, taken from the center of the loaf, was pressed to reach 50% height by a P/36 aluminum compression plate in two cycles with a 5 s delay. The obtained TPA parameters (hardness, cohesiveness, chewiness, resilience of the crumb) were used as indicators of textural properties. The calculations were performed using the attached software Texture Exponent (Stable Microsystems, Godalming, UK).

### 3.12. Quantitative Descriptive Analysis

Quantitative descriptive analysis (QDA) according to ISO 13299:2016 [63] of the prepared breads was carried out by an 11-person panel (nine women and two men) with verified sensory sensitivity [64]. In the first stage, the assessors familiarized themselves with the product, which was bread, and under the guidance of the leader, selected appropriate descriptors and a rating scale for the product. Then, they verified the prepared list by means of a trial assessment of selected breads. The proper assessment included a set of four coded samples, presented in the form of a 1.5 cm thick slice in random order. Spring water was used to rinse the mouth between assessments of subsequent samples. The assessment included smell, crust characteristics, crumb characteristics, and taste (Table 8), and the intensity of the observed impressions was presented on an unstructured linear scale of 10 cm with established boundary terms.

### 3.13. Consumer Acceptance Analysis

The assessment of product acceptance was carried out using a 10-point Likert scale. The overall assessment included the smell of the products, their taste, the appearance and structure of the crust, the appearance and structure of the crumb, and the overall quality of the product. The data were presented in the form of average results and standard deviations, as well as in the form of radar charts. All participants voluntarily signed consent forms for participation in the study, which was approved by the Independent Bioethics Committee for Research of the Medical University of Gdańsk (KB/336/2023). This study is in line with the ethical principles of non-violence, beneficence, justice, and autonomy contained in the ethical provisions of the 2013 revised Declaration of Helsinki.

### 3.14. Statistical Analysis

Results are expressed as arithmetic mean ± standard deviation. The significance of differences was demonstrated by analysis of variance at a significance level of *p* ≤ 0.05 and Fisher’s post hoc test. Calculations were performed using Statistica 13.0 software (Tibco Software Inc., Palo Alto, CA, USA).

K-means clustering was employed to group similar bread samples based on their taste and smell attributes into distinct clusters. The elbow method was applied to determine the optimal number of clusters (k) for the K-means clustering algorithm. The point where the rate of decrease sharply diminishes, known as the “elbow”, indicates the optimal number of clusters. In this case, the elbow point suggests that the most appropriate number of clusters was k = 3 (Figure S4). Principal Component Analysis (PCA) was used to reduce the data to two principal components, which act as a compressed representation of the original features. Clustering and Principal Component Analysis was conducted using Python version 3.10.12.

## 4. Conclusions

The conducted studies indicated that the use of fermentation affects the change in sensory properties and consumer acceptance of hemp bread. The best-rated breads were those with the addition of wheat and wheat-hemp sourdough. This was probably the result of better acidification (observed as a decrease in the pH value of sourdoughs over time), which contributed to the formation of flavor and aroma compounds that improve the sensory properties of the obtained products. Both the quantitative descriptive analysis and the statistical modeling confirm the above observations. The performed studies suggest that the use of fermentation can improve the final quality of breads with the addition of hemp flour, but further studies are required to increase the degree of acidification of hemp flour. It is possible to use other fermentation starters or apply different fermentation conditions.

## Figures and Tables

**Table 1:** Chemical composition of basic raw materials and bread (g/100 g d.m.).

Sample	Water Content	Protein	Fat	Ash	Insoluble Fiber	Soluble Fiber	Total Fiber	Carbohydrate
	[g/100 g d.m.]							
WF	13.34 ^b^ ± 0.16	13.18 ^a^ ± 0.13	1.47 ^a^ ± 0.00	0.67 ^a^ ± 0.02	1.59 ^a^ ± 0.02	1.31 ^a^ ± 0.04	2.90 ^a^ ± 0.07	81.78 ^b^ ± 0.04
HF	8.08 ^a^ ± 0.04	28.01 ^b^ ± 0.98	12.71 ^b^ ± 0.08	6.86 ^b^ ± 0.02	42.70 ^b^ ± 0.15	8.21 ^b^ ± 0.12	50.91 ^b^ ± 0.03	1.49 ^a^ ± 0.13
HB1	7.40 ^ab^ ± 0.01	16.10 ^a^ ± 0.25	2.87 ^a^ ± 0.10	3.41 ^a^ ± 0.02	6.54 ^a^ ± 0.03	2.42 ^a^ ± 0.02	8.97 ^b^ ± 0.05	68.65 ^a^ ± 0.13
HB2	7.32 ^a^ ± 0.08	15.72 ^a^ ± 0.20	2.76 ^a^ ± 0.08	3.36 ^a^ ± 0.05	6.44 ^a^ ± 0.11	2.44 ^a^ ± 0.05	8.87 ^ab^ ± 0.07	69.19 ^a^ ± 0.19
HB3	7.37 ^ab^ ± 0.03	15.85 ^a^ ± 0.27	2.94 ^a^ ± 0.11	3.36 ^a^ ± 0.03	6.33 ^a^ ± 0.11	2.37 ^a^ ± 0.05	8.70 ^a^ ± 0.05	69.15 ^a^ ± 0.19
HB4	7.48 ^b^ ± 0.06	15.80 ^a^ ± 0.22	2.75 ^a^ ± 0.10	3.35 ^a^ ± 0.01	6.45 ^a^ ± 0.03	2.40 ^a^ ± 0.16	8.86 ^ab^ ± 0.13	69.25 ^a^ ± 0.02

Explanatory notes: WF—wheat flour; HF—hemp flour; HB1—hemp bread; HB2—hemp bread with wheat sourdough; HB3—hemp bread with wheat and hemp sourdough; HB4—hemp bread with hemp sourdough; values at the same column marked with different letters are statistically significantly different at *p* ≤ 0.05 ± SD.

**Table 2:** Evaluation of the physical properties of the tested breads.

Bread Sample	Volume (mL)	Weight (g)	Specific Volume (mL/g)	Volume-Yield (mL/100 g Flour)
HB1	672 ^b^ ± 12	213 ^a^ ± 3	3.16 ^c^ ± 0.08	457 ^b^ ± 8
HB2	614 ^a^ ± 11	215 ^ab^ ± 2	2.86 ^a^ ± 0.06	418 ^a^ ± 7
HB3	629 ^a^ ± 6	214 ^a^ ± 3	2.95 ^b^ ± 0.05	428 ^a^ ± 4
HB4	618 ^a^ ± 18	217 ^b^ ± 2	2.85 ^a^ ± 0.07	420 ^a^ ± 12

Explanatory notes: HB1—hemp bread; HB2—hemp bread with wheat sourdough; HB3—hemp bread with wheat and hemp sourdough; HB4—hemp bread with hemp sourdough; values at the same column marked with different letters are statistically significantly different at *p* ≤ 0.05 ± SD.

**Table 3:** The color of the bread crumb.

Bread Sample	L* (D65)	a* (D65)	b* (D65)	ΔE
HB1	42.70 ^b^ ± 0.92	3.02 ^a^ ± 0.20	14.46 ^a^ ± 0.63	-
HB2	40.76 ^a^ ± 0.42	4.09 ^d^ ± 0.06	18.31 ^bc^ ± 0.26	4.46 ^b^ ± 0.25
HB3	43.71 ^b^ ± 1.73	3.59 ^c^ ± 0.15	18.69 ^c^ ± 0.23	4.64 ^b^ ± 0.61
HB4	42.19 ^b^ ± 1.16	3.33 ^b^ ± 0.06	18.02 ^b^ ± 0.31	3.76 ^a^ ± 0.34

Explanatory notes: HB1—hemp bread; HB2—hemp bread with wheat sourdough; HB3—hemp bread with wheat and hemp sourdough; HB4—hemp bread with hemp sourdough; values at the same column marked with different letters are statistically significantly different at *p* ≤ 0.05 ± SD.

**Table 4:** Breads moisture and texture parameter changes during storage.

Bread Sample	Day of Analysis	Hardness [N]	Cohesiveness [-]	Chewiness [N]	Resilience [-]	Moisture Content [%]
HB1	0	15.38 ^ab^ ± 2.14	0.765 ^e^ ± 0.070	11.1 ^a^ ± 1.3	0.424 ^d^ ± 0.051	42.45 ^a^ ± 0.25
HB2	0	16.23 ^abc^ ± 1.89	0.708 ^d^ ± 0.021	11.0 ^a^ ± 1.4	0.397 ^d^ ± 0.019	44.28 ^bcde^ ± 0.66
HB3	0	15.20 ^ab^ ± 2.70	0.748 ^de^ ± 0.009	10.8 ^a^ ± 2.0	0.425 ^d^ ± 0.010	44.17 ^bcde^ ± 0.93
HB4	0	13.63 ^a^ ± 2.57	0.736 ^de^ ± 0.010	9.6 ^a^ ± 1.6	0.418 ^d^ ± 0.012	45.01 ^e^ ± 0.53
HB1	1	22.70 ^abcd^ ± 5.41	0.609 ^c^ ± 0.031	13.0 ^ab^ ± 3.1	0.297 ^c^ ± 0.026	44.99 ^e^ ± 0.47
HB2	1	22.07 ^abcd^ ± 4.60	0.628 ^c^ ± 0.021	12.9 ^ab^ ± 2.6	0.311 ^c^ ± 0.017	44.56 ^de^ ± 0.40
HB3	1	26.17 ^bcde^ ± 2.70	0.637 ^c^ ± 0.030	15.6 ^ab^ ± 1.6	0.321 ^c^ ± 0.027	43.95 ^bcde^ ± 0.69
HB4	1	22.55 ^abcd^ ± 2.38	0.632 ^c^ ± 0.030	13.4 ^ab^ ± 1.7	0.316 ^c^ ± 0.022	44.74 ^de^ ± 0.77
HB1	2	27.09 ^cde^ ± 5.85	0.551 ^ab^ ± 0.017	13.9 ^ab^ ± 3.4	0.248 ^b^ ± 0.016	44.46 ^cde^ ± 0.32
HB2	2	28.29 ^de^ ± 8.35	0.521 ^ab^ ± 0.020	13.8 ^ab^ ± 4.4	0.225 ^ab^ ± 0.014	43.23 ^ab^ ± 0.13
HB3	2	31.52 ^de^ ± 9.16	0.550 ^ab^ ± 0.031	16.5 ^ab^ ± 5.2	0.247 ^b^ ± 0.025	43.74 ^bcd^ ± 0.21
HB4	2	34.74 ^e^ ± 14.78	0.560 ^b^ ± 0.032	18.2 ^b^ ± 8.4	0.251 ^b^ ± 0.023	44.49 ^cde^ ± 0.05
HB1	3	27.37 ^cde^ ± 8.10	0.525 ^ab^ ± 0.017	13.4 ^ab^ ± 4.4	0.224 ^ab^ ± 0.018	44.66 ^de^ ± 0.20
HB2	3	33.13 ^de^ ± 10.66	0.505 ^a^ ± 0.030	16.0 ^ab^ ± 6.0	0.204 ^a^ ± 0.019	43.36 ^abc^ ± 0.13
HB3	3	30.50 ^de^ ± 6.39	0.522 ^ab^ ± 0.043	15.1 ^ab^ ± 4.2	0.223 ^ab^ ± 0.032	44.60 ^de^ ± 0.10
HB4	3	27.97 ^cde^ ± 10.73	0.554 ^ab^ ± 0.027	14.7 ^ab^ ± 6.3	0.250 ^b^ ± 0.016	45.04 ^e^ ± 0.55

Explanatory notes: HB1—hemp bread; HB2—hemp bread with wheat sourdough; HB3—hemp bread with wheat and hemp sourdough; HB4—hemp bread with hemp sourdough; values at the same column marked with different letters are statistically significantly different at *p* ≤ 0.05 ± SD.

**Table 5:** Amino acid (AA) profile of wheat and wheat-hempseed bread (mg/g of protein).

	WF	HF	HB1	HB2	HB3	HB4
Amino acid	Essential amino acids (EAA)					
Histidine	22.67 ^a^ ± 0.23	22.74 ^a^ ± 0.63	24.45 ^a^ ± 0.23	24.55 ^a^ ± 0.25	24.24 ^a^ ± 0.21	24.18 ^a^ ± 0.21
Isoleucine	33.86 ^a^ ± 0.42	36.28 ^b^ ± 0.40	36.20 ^a^ ± 0.50	36.34 ^a^ ± 0.47	35.65 ^a^ ± 0.62	35.42 ^a^ ± 0.66
Leucine	65.72 ^b^ ± 0.89	60.79 ^a^ ± 0.61	66.73 ^b^ ± 0.96	66.73 ^b^ ± 0.76	65.57 ^ab^ ± 1.09	64.81 ^a^ ± 0.99
Lysine	21.99 ^a^ ± 0.20	35.01 ^b^ ± 0.43	27.52 ^a^ ± 0.86	27.66 ^a^ ± 0.28	26.99 ^a^ ± 0.43	27.08 ^a^ ± 0.55
Methionine	18.50 ^a^ ± 0.93	25.24 ^b^ ± 1.19	18.49 ^b^ ± 0.26	16.94 ^a^ ± 0.37	18.27 ^b^ ± 1.13	18.13 ^b^ ± 0.16
Phenylalanine	47.00 ^b^ ± 0.55	42.60 ^a^ ± 0.53	48.02 ^a^ ± 1.01	48.28 ^a^ ± 0.55	47.48 ^a^ ± 0.83	47.29 ^a^ ± 0.73
Threonine	25.63 ^a^ ± 0.21	31.62 ^b^ ± 0.38	29.13 ^b^ ± 0.52	29.22 ^b^ ± 0.29	28.80 ^ab^ ± 0.44	28.26 ^a^ ± 0.37
Valine	38.91 ^a^ ± 0.44	45.23 ^b^ ± 0.55	42.97 ^b^ ± 0.65	43.12 ^b^ ± 0.54	42.33 ^ab^ ± 0.67	41.95 ^a^ ± 0.45
Total EAA	274.28 ^a^ ± 3.13	299.51 ^b^ ± 2.42	293.51 ^b^ ± 4.43	292.84 ^b^ ± 3.32	289.31 ^ab^ ± 4.54	287.11 ^a^ ± 3.81
	Non-essential amino acids (non-EAA)					
Alanine	29.24 ^a^ ± 0.21	39.50 ^b^ ± 0.45	34.85 ^a^ ± 0.57	35.05 ^a^ ± 0.34	34.65 ^a^ ± 0.59	34.51 ^a^ ± 0.40
Arginine	37.26 ^a^ ± 0.63	108.89 ^b^ ± 1.84	61.60 ^b^ ± 1.31	61.11 ^ba^ ± 1.14	60.55 ^ba^ ± 1.16	59.23 ^a^ ± 1.37
Aspartic acid	41.52 ^a^ ± 0.82	96.20 ^b^ ± 1.84	60.90 ^a^ ± 0.89	61.10 ^a^ ± 0.83	60.85 ^a^ ± 1.08	60.80 ^a^ ± 0.90
Cysteine	22.90 ^b^ ± 0.18	16.68 ^a^ ± 0.72	21.07 ^c^ ± 0.26	19.44 ^a^ ± 0.29	19.81 ^ab^ ± 0.69	20.34 ^b^ ± 0.06
Glutamic acid	356.05 ^b^ ± 3.96	166.61 ^a^ ± 1.96	301.31 ^b^ ± 4.73	303.15 ^b^ ± 3.30	295.12 ^ab^ ± 4.96	291.45 ^a^ ± 4.02
Glycine	35.07 ^a^ ± 0.26	40.72 ^b^ ± 0.46	38.60 ^a^ ± 0.65	38.71 ^a^ ± 0.43	38.01 ^ab^ ± 0.56	37.80 ^a^ ± 0.46
Proline	113.04 ^b^ ± 1.15	33.40 ^a^ ± 0.43	89.62 ^b^ ± 1.25	90.41 ^b^ ± 1.09	88.65 ^ab^ ± 1.83	86.72 ^a^ ± 1.34
Serine	46.66 ^b^ ± 0.43	45.60 ^a^ ± 0.60	48.31 ^a^ ± 0.82	48.28 ^a^ ± 0.41	47.23 ^a^ ± 0.71	46.45 ^a^ ± 0.64
Tyrosine	24.37 ^a^ ± 0.95	28.28 ^b^ ± 0.38	27.68 ^a^ ± 1.42	26.19 ^a^ ± 0.41	26.84 ^a^ ± 0.91	26.33 ^a^ ± 0.49
Total non-EAA	706.11 ^b^ ± 7.30	575.88 ^a^ ± 6.49	683.94 ^b^ ± 11.31	683.44 ^b^ ± 7.86	671.72 ^ab^ ± 11.72	663.63 ^a^ ± 9.44

Explanatory notes: WF—wheat flour; HF—hemp flour; HB1—hemp bread; HB2—hemp bread with wheat sourdough; HB3—hemp bread with wheat and hemp sourdough; HB4—hemp bread with hemp sourdough; values at the same row marked with different letters are statistically significantly different at *p* ≤ 0.05 ± SD.

**Table 6:** Nutritional value of protein of wheat and wheat-hempseed bread.

		AAS [%]					
EAA	FAO 2011 Reference	WF	HF	HB1	HB2	HB3	HB4
Val	40.00	97.28 ^a^	113.08 ^b^	107.44 ^b^	107.79 ^b^	105.81 ^a^	104.88 ^a^
Thr	25.00	102.50 ^a^	126.48 ^b^	116.52 ^b^	116.89 ^b^	115.20 ^b^	113.05 ^a^
Ile	30.00	112.86 ^a^	120.93 ^b^	120.67 ^b^	121.12 ^b^	118.82 ^a^	118.07 ^a^
His	16.00	141.68 ^a^	142.13 ^a^	152.84 ^a^	153.42 ^a^	151.48 ^a^	151.10 ^a^
Leu	61.00	107.74 ^b^	99.65 ^a^	109.39 ^b^	109.40 ^b^	107.49 ^a^	106.24 ^a^
Lys	48.00	45.82 ^a^	72.93 ^b^	57.34 ^a^	57.63 ^a^	56.22 ^a^	56.42 ^a^
AAA	41.00	174.08 ^b^	172.88 ^a^	184.63 ^b^	181.65 ^a^	181.27 ^b^	179.55 ^a^
SAA	23.00	179.98 ^a^	182.25 ^b^	171.96 ^c^	158.18 ^a^	165.55 ^b^	167.23 ^b^

Explanatory notes: WF—wheat flour; HF—hemp flour; HB1—hemp bread; HB2—hemp bread with wheat sourdough; HB3—hemp bread with wheat and hemp sourdough; HB4—hemp bread with hemp sourdough; SAA—Sulphur-containing AA; AAA—aromatic AA; values at the same column marked with different letters are statistically significantly different at *p* ≤ 0.05 ± SD.

**Table 7:** Fatty acids profile of hemp and wheat flour and obtained breads.

Fatty Acid	WF *	HF	HB1	HB2	HB3	HB4
C14:0	0.09 ± 0.00	n.d.	n.d.	n.d.	0.12 ± 0.00	n.d.
C16:0	17.62 ^B^** ± 0.00	7.02 ^A^ ± 0.06	7.74 ^a^ ± 0.02	8.64 ^b^ ± 0.18	10.72 ^c^ ± 0.04	8.74 ^b^ ± 0.02
C16:1	0.12 ^A^ ± 0.00	0.13 ^B^ ± 0.00	0.36 ^a^ ± 0.00	0.37 ^b^ ± 0.01	0.39 ^c^ ± 0.00	0.37 ^b^ ± 0.00
C18:0	0.69 ^A^ ± 0.00	2.50 ^B^ ± 0.03	2.34 ^a^ ± 0.01	2.58 ^c^ ± 0.02	3.18 ^d^ ± 0.00	2.48 ^b^ ± 0.00
C18:1 cis	9.18 ^A^ ± 0.00	10.05 ^B^ ± 0.00	31.47 ^d^ ± 0.21	24.60 ^c^ ± 0.11	17.89 ^a^ ± 0.05	21.17 ^b^ ± 0.02
C18:1 trans	n.d.	1.00 ± 0.00	1.88 ^d^ ± 0.01	1.60 ^c^ ± 0.01	1.24 ^a^ ± 0.01	1.45 ^b^ ± 0.02
C18:2 cis	n.d.	55.33 ± 0.05	40.29 ^a^ ± 0.14	45.16 ^b^ ± 0.24	49.33 ^d^ ± 0.15	48.26 ^c^ ± 0.09
C18:2 trans	n.d.	0.08 ± 0.05	0.09 ^a^ ± 0.02	0.06 ^a^ ± 0.01	0.12 ^a^ ± 0.02	0.13 ^a^ ± 0.04
C18:3 n-6	67.25 ^B^ ± 0.00	4.36 ^A^ ± 0.01	2.34 ^a^ ± 0.01	2.82 ^b^ ± 0.01	3.11 ^c^ ± 0.00	3.10 ^c^ ± 0.00
C18:3 n-3	4.16 ^A^ ± 0.00	17.17 ^B^ ± 0.09	10.54 ^a^ ± 0.04	10.95 ^b^ ± 0.07	11.11 ^c^ ± 0.05	11.53 ^d^ ± 0.02
C20:0	0.07 ^A^ ± 0.00	1.01 ^B^ ± 0.01	0.97 ^a^ ± 0.17	1.07 ^a^ ± 0.14	0.96 ^a^ ± 0.04	0.92 ^a^ ± 0.01
C22:6	n.d.	0.55 ± 0.01	0.97 ^c^ ± 0.14	0.89 ^c^ ± 0.10	0.67 ^a^ ± 0.05	0.75 ^b^ ± 0.00
C20:2	n.d.	0.11 ± 0.00	0.22 ^a^ ± 0.11	0.24 ^a^ ± 0.09	0.21 ^a^ ± 0.06	0.18 ^a^ ± 0.02
C22:0	n.d.	0.48 ± 0.00	0.53 ^a^ ± 0.01	0.62 ^c^ ± 0.00	0.60 ^c^ ± 0.01	0.59 ^b^ ± 0.00
C24:0	n.d.	0.23 ± 0.01	0.26 ^a^ ± 0.01	0.28 ^a^ ± 0.03	0.27 ^a^ ± 0.00	0.34 ^b^ ± 0.04
SFA	18.65 ^B^ ± 0.00	11.23 ^A^ ± 0.11	11.83 ^a^ ± 0.12	13.20 ^b^ ± 0.27	15.85 ^c^ ± 0.02	13.06 ^b^ ± 0.07
MUFA	9.93 ^A^ ± 0.00	11.17 ^B^ ± 0.08	33.71 ^d^ ± 0.20	26.56 ^c^ ± 0.12	19.53 ^a^ ± 0.04	23.00 ^b^ ± 0.05
PUFA	71.42 ^A^ ± 0.00	77.60 ^B^ ± 0.19	54.46 ^a^ ± 0.08	60.11 ^b^ ± 0.13	64.55 ^c^ ± 0.08	63.95 ^c^ ± 0.12
PUFA/SFA	3.83 ^A^ ± 0.07	6.91 ^B^ ± 0.08	4.60 ^b^ ± 0.04	4.56 ^b^ ± 0.10	4.07 ^a^ ± 0.01	4.90 ^c^ ± 0.04
n-6	67.25 ^B^ ± 0.00 a	59.88 ^A^ ± 0.11	42.94 ^a^ ± 0.02	48.27 ^b^ ± 0.17	52.77 ^d^ ± 0.07	51.66 ^c^ ± 0.13
n-3	4.16 ^A^ ± 0.00 c	17.71 ^B^ ± 0.08	11.51 ^a^ ± 0.10	11.84 ^b^ ± 0.03	11.78 ^b^ ± 0.01	12.28 ^c^ ± 0.02
n-6/n-3	16.15 ^B^ ± 0.09	3.38 ^A^ ± 0.01	3.73 ^a^ ± 0.03	4.08 ^b^ ± 0.03	4.48 ^d^ ± 0.00	4.21 ^c^ ± 0.02

Explanatory notes: WF—wheat flour; HF—hemp flour; HB1—hemp bread; HB2—hemp bread with wheat sourdough; HB3—hemp bread with wheat and hemp sourdough; HB4—hemp bread with hemp sourdough; n.d.—not detected; * according to Kowalski et al. [44]; ** values in rows marked with the same capital letter for flours and with a lower-case letter for breads do not differ statistically significantly at *p* ≤ 0.05.

**Table 8:** Qualitative features, definitions, and boundary terms used in the descriptive analysis.

Quality Features	Definition	Boundary Terms (0–10 c.u.)
Smell		
sour	aroma associated with sour substances	imperceptible—very intense
nutty	aroma characteristic of a nut mixture	
mill	aroma characteristic of the mill	
earthy	an aroma typical of wet earth	
maritime	aroma typical of the sea	
Crust features		
brownness	combination of green and red in different proportions; e.g., RGB (120, 67, 21)	imperceptible—very intense
greyness	combination of white and black in different proportions; e.g., RGB (161, 161, 161)	
hardness	the force required to deform the product, felt when squeezing the product between the teeth	imperceptible—very intense
crispness	when biting or breaking the product, a dry, crackling sound is heard	
Crumb features		
brownness	combination of green and red in different proportions; e.g., RGB (120, 67, 21)	imperceptible—very intense
greyness	combination of white and black in different proportions; e.g., RGB (161, 161, 161)	
greenness	combination of yellow and blue in various proportions; e.g., RGB (70, 158, 43)	
porosity	determines the amount of empty space inside the product, visible in the cross-section	imperceptible—very intense
graininess	the amount of fine particles in the chewed mass	imperceptible—very intense
Taste		
bitter	the degree of perception of bitter taste as a basic taste	imperceptible—very intense
sour	degree of perception of sour taste as a basic taste	
malty	the degree of perceived aromatic flavor associated with malt	
cereal	the degree of perceived flavor aromaticity associated with cereal flakes	

## Data Availability

Data are contained within the article and Supplementary Materials.

## Supplementary Materials

The following supporting information can be downloaded at: https://www.mdpi.com/article/10.3390/molecules29225455/s1, Figure S1. Change of sourdough pH during fermentation (SW—wheat flour sourdough), SH—hemp flour sourdough, SHW—sourdough made from wheat and hemp flour in a 50/50 ratio); Figure S2. Protein profiles obtained by SDS-PAGE electrophoretic separation. Left edge—molecular weight markers, 1—HB1; 2—HB2; 3—HB3; 4—HB4; 5—wheat flour; 6—wheat sourdough; 7—mixed sourdough; 8—hemp sourdough; 9—hemp flour; Figure S3. Sensory profile of bread with the addition of/participation of hemp flour; Figure S4. Determine the optimal number of clusters (k) for K-means clustering; Table S1. Results of e-nose and e-tongue analysis of sourdoughs used to prepare breads; Table S2. E-nose and e-tongue analysis results of hemp bread; Table S3. Mean values of selected features for every cluster.
