# Supplementary material for: The Influence of Fermentation Technology on the Functional and Sensory Properties of Hemp Bread
Source: Molecules. 2024 Nov 19;29(22):5455. doi: 10.3390/molecules29225455 (PMC11597250; doi:10.3390/molecules29225455)
Supplement: Supplementary file 1 [file molecules-29-05455-s001.zip › molecules-3297980-supplementary.pdf]

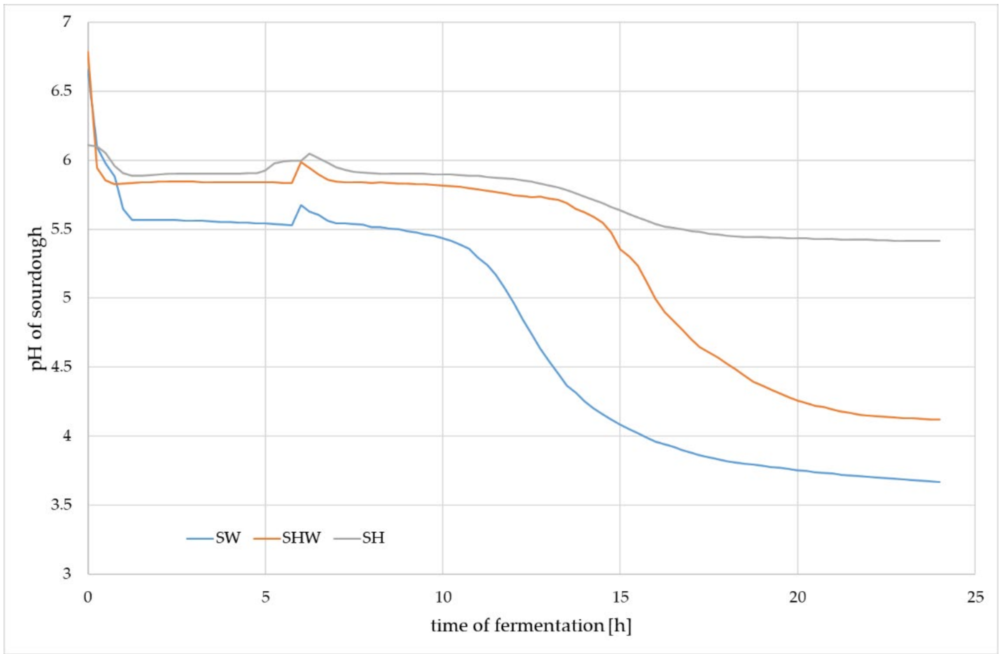

Figure S1. Change of sourdough pH during fermentation (SW - wheat flour sourdough), SH - hemp flour sourdough, SHW - sourdough made from wheat and hemp flour in a 50/50 ratio)

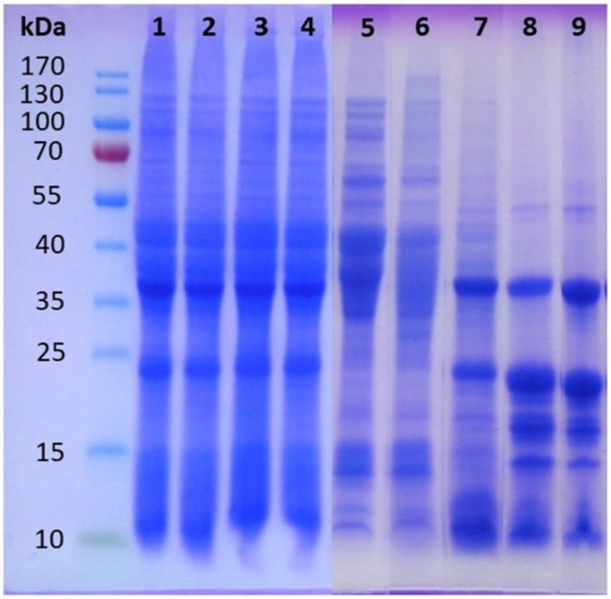

Figure S2. Protein profiles obtained by SDS-PAGE electrophoretic separation. Left edge - molecular weight markers, 1 - HB1; 2 - HB2; 3 - HB3; 4 - HB4; 5 - wheat flour; 6 - wheat sourdough; 7 - mixed sourdough; 8 - hemp sourdough; 9 - hemp flour

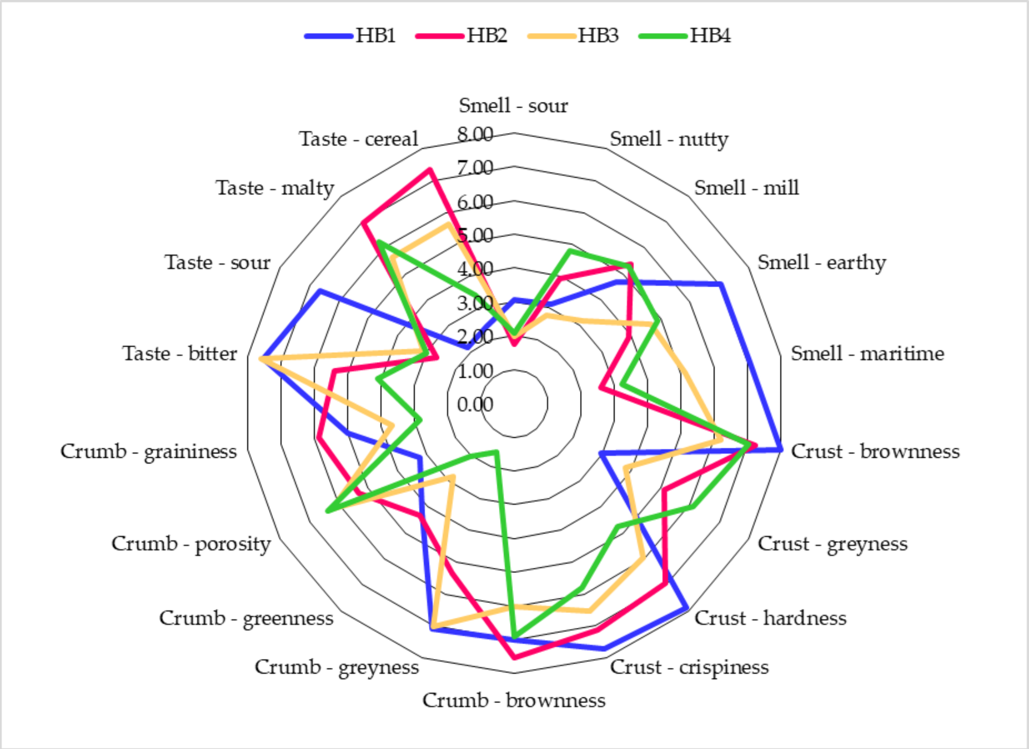

Figure S3. Sensory profile of bread with the addition of/participation of hemp flour

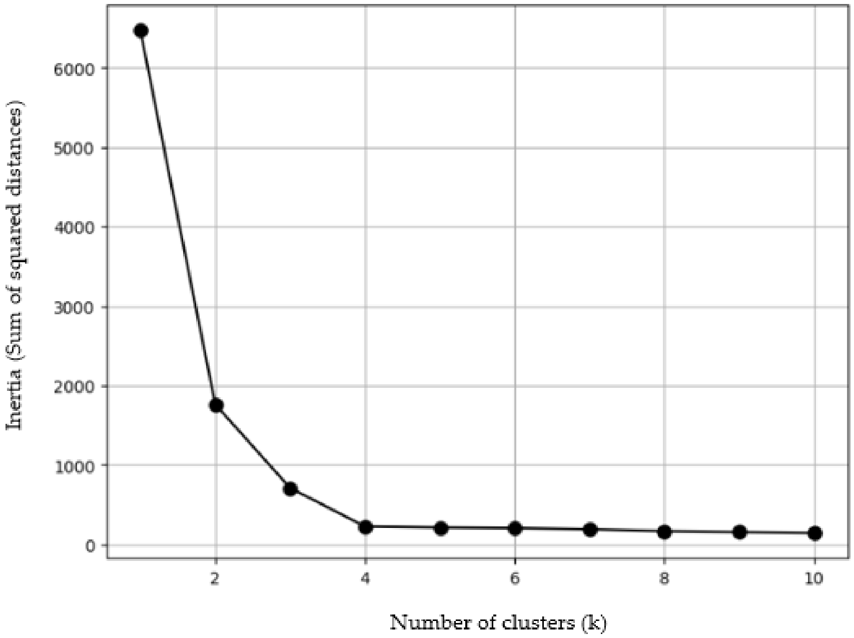

Figure S4. Determine the optimal number of clusters (k) for K-means clustering

Table S1 Results of e-nose and e-tongue analysis of sourdoughs used to prepare breads

	SH	SHW	SW
SRS	3.5 0±0.141b	3.45 ±0.071b	7.85 ±0.212a
GPS	5.200±0.140a	5.200±0.000a	5.0 0±0.141a
STS	9.425±0.389a	4.650±0.071b	8.850±0.073a
UMS	3.015±0.163c	8.650±0.071b	9.575±0.106a
SPS	8.800±0.141a	4.150±0.212b	2.300±0.139c
SWS	8.450±0.071a	8.525±0.035a	5.465±0.092b
BRS	4.200±0.141b	5.225±0.036a	2.300±0.141c
Acetaldehyde	7.857±0.028c	8.006±0.013b	8.073±0.015a
2-methylpropanal	3.961±0.014c	4.036±0.006b	4.070±0.007a
Ethanol	0.190±0.001c	0.193±0.000b	0.195±0.001a
Butanal	36.922±0.131c	37.620±0.059b	37.935±0.060a
Butane-2,3-dione	2.967±0.011c	3.023±0.005b	3.048±0.004a
Acetic acid	0.446±0.002c	0.455±0.001b	0.458±0.001a
Ethyl Acetate	3.987±0.014c	4.062±0.006b	4.096±0.007a
3-Methylbutanal	25.162±0.089c	25.638±0.040b	25.852±0.041a
n-butanol	0.439±0.002a	0.514±0.093a	0.518±0.094a
2,3-Pentanedione	0.173±0.001c	0.176±0.000b	0.178±0.000a
Propanoic acid	1.356±0.005c	1.381±0.002b	1.393±0.002a
Methyl 2-methylbutanoate	0.117±0.000c	0.120±0.000b	0.121±0.000a
Butanoic acid	0.168±0.001c	0.171±0.000b	0.172±0.000a
Butyl acetate	1.488±0.005a	1.561±0.061a	1.574±0.062a
1-Hexanol	2.978±0.011c	3.035±0.005b	3.060±0.005a
Hexanoic acid	0.772±0.003c	0.787±0.001b	0.794±0.001a
Trimethylpyrazine	1.114±0.004c	1.135±0.002b	1.144±0.002a
Benzeneacetaldehyde	0.126±0.000c	0.129±0.000b	0.130±0.000a
1-Octanol	0.154±0.001c	0.157±0.000b	0.158±0.000a
Benzaldehyde	1.287±0.005a	0.580±0.001b	0.234±0.000c
Nonan-2-one	0.177±0.001c	0.181±0.000b	0.182±0.000a
2-Phenylethanol	0.458±0.002c	0.467±0.001b	0.471±0.001a
Maltol	0.251±0.001c	0.255±0.000b	0.257±0.000a
Ethyl malto	0.365±0.001c	0.372±0.001b	0.375±0.001a
4-ethylguaiacol	0.038±0.000c	0.039±0.000b	0.040±0.000a
gamma-Nonalactone	1.076±0.004a	0.467±0.001b	0.169±0.000c
2-Phenylethyl acetate	3.626±0.013c	3.695±0.006b	3.726±0.006a
delta-Decalactone	0.381±0.001c	0.388±0.001b	0.392±0.001a
2-Acetylfuran	0.217±0.001c	0.221±0.000b	0.223±0.000a
Benzyl benzoate	0.866±0.003a	0.496±0.001b	0.314±0.000c

Ethyl tetradecanoate	0.177±0.001c	0.181±0.000b	0.182±0.000a
Ethyl tetradecanoate;Benzyl phenyl acetate	0.162±0.001c	0.165±0.000b	0.166±0.000a
Octyl acetate	0.291±0.001c	0.297±0.000b	0.299±0.000a

Explanatory notes:
SH – hemp sourdough, SW – wheat sourdough, SHW – hemp-wheat sourdough
SRS (sourness), GPS (metallic), STS (saltiness), SPS (spiciness), UMS (umami), SWS (sweetness), and BRS (bitterness)

Table S2 E-nose and e-tongue analysis results of hemp bread

	HB1	HB2	HB3	HB4
SRS	3.50±0.13b	7.45±0.07a	7.57±0.10a	7.50±0.15a
GPS	5.35±0.07b	11.80±0.15a	5.30±0.14b	5.25±0.07b
STS	4.40±0.14b	4.60±0.14b	5.07±0.09a	5.20±0.15a
UMS	4.90±0.15b	5.00±0.13b	5.60±0.12a	5.20±0.12b
SPS	8.20±0.12a	6.90±0.14b	6.20±0.13c	6.50±0.16c
SWS	4.00±0.14c	4.08±0.11bc	4.37±0.05ab	4.50±0.13a
BRS	5.95±0.07d	6.82±0.12c	7.40±0.14b	8.00±0.14a
Acetaldehyde	2.80±0.01b	2.60±0.01c	3.16±0.01a	2.16±0.01d
2-methylpropanal	1.41±0.01a	1.42±0.01a	1.41±0.01a	0.97±0.00b
Ethanol	66.96±0.24b	67.31±0.24b	66.93±0.24b	77.29±0.27a
Butanal	6.40±0.02a	6.43±0.02a	6.40±0.02a	4.38±0.02b
Butane-2,3-dione	1.06±0.00a	1.06±0.00a	1.06±0.00a	0.72±0.00b
Acetic acid	0.16±0.00a	0.15±0.00b	0.15±0.00b	0.10±0.00c
Ethyl Acetate	1.42±0.01a	1.43±0.01a	1.42±0.01a	0.97±0.00b
3-Methylbutanal	8.97±0.03a	9.02±0.03a	8.97±0.03a	6.14±0.02b
n-butanol	4.21±0.01a	4.23±0.01a	4.21±0.01a	2.88±0.01b
2,3-Pentanedione	0.06±0.00a	0.06±0.00a	0.06±0.00a	0.04±0.00b
Propanoic acid	0.48±0.00a	0.49±0.00a	0.48±0.00a	0.33±0.00b
Methyl 2-methylbutanoate	0.04±0.00a	0.04±0.00a	0.04±0.00a	0.03±0.00b
Butanoic acid	0.06±0.00a	0.06±0.00a	0.06±0.00a	0.04±0.00b
Butyl acetate	0.56±0.00a	0.57±0.00a	0.56±0.00a	0.38±0.00b
1-Hexanol	1.06±0.00a	0.85±0.00b	0.84±0.00b	0.58±0.00c
Hexanoic acid	0.28±0.00a	0.28±0.00a	0.28±0.00a	0.19±0.00b
Trimethylpyrazine	0.40±0.00a	0.40±0.00a	0.40±0.00a	0.27±0.00b
Benzeneacetaldehyde	0.05±0.00a	0.05±0.00a	0.05±0.00a	0.03±0.00b
1-Octanol	0.05±0.00a	0.06±0.00a	0.05±0.00a	0.04±0.00b
Benzaldehyde	0.47±0.00a	0.41±0.00b	0.41±0.00b	0.28±0.00c
Nonan-2-one	0.06±0.00a	0.06±0.00a	0.06±0.00a	0.04±0.00b
2-Phenylethanol	0.16±0.00a	0.16±0.00a	0.16±0.00a	0.11±0.00b

Maltol	0.09±0.00a	0.07±0.00b	0.07±0.00b	0.05±0.00c
Ethyl malto	0.13±0.00a	0.13±0.00a	0.13±0.00a	0.09±0.00b
4-ethylguaiacol	0.50±0.00a	0.50±0.00a	0.50±0.00a	0.34±0.00b
gamma-Nonalactone	0.06±0.00a	0.06±0.00a	0.06±0.00a	0.04±0.00b
2-Phenylethyl acetate	1.29±0.00a	1.30±0.00a	1.29±0.00a	0.88±0.00b
delta-Decalactone	0.14±0.00a	0.14±0.00a	0.14±0.00a	0.09±0.00b
2-Acetylfuran	0.08±0.00a	0.08±0.00a	0.08±0.00a	0.05±0.00b
Benzyl benzoate	0.11±0.00a	0.11±0.00a	0.11±0.00a	0.07±0.00b
Ethyl tetradecanoate	0.06±0.00a	0.06±0.00a	0.06±0.00a	0.04±0.00b
Ethyl tetradecanoate;Benzyl phenyl acetate	0.06±0.00a	0.06±0.00a	0.06±0.00a	0.04±0.00b
Octyl acetate	0.10±0.00a	0.10±0.00a	0.10±0.00a	0.07±0.00b

Explanatory notes:
SH – hemp sourdough, SW – wheat sourdough, SHW – hemp-wheat sourdough
SRS (sourness), GPS (metallic), STS (saltiness), SPS (spiciness), UMS (umami), SWS (sweetness), and BRS (bitterness)

Table S3. Mean values of selected features for every cluster

Attribute			Cluster		
			**0**	**1**	**2**
**Device assesment**	**taste**	SRS	7.540	7.500	**3.500**
		GPS	**8.583**	5.300	5.400
		SPS	6.583	6.533	**8.233**
		BRS	7.122	8.033	6.000
	**smell**	2-methylpropanal	1.416	**0.966**	1.414
		Ethanol	67.121	**77.224**	67.012
		Butanal	6.414	**4.373**	6.404
		Butane-2,3-dione	1.061	**0.723**	1.059
		Acetic acid	0.151	**0.103**	0.159
		Ethyl Acetate	1.425	**0.972**	1.423
		3-Methylbutanal	8.997	**6.134**	8.982
		n-butanol	4.217	**2.875**	4.210
		1-Hexanol	0.844	0.575	1.063
		Hexanoic acid	0.276	**0.188**	0.276
		1-Octanol	0.055	**0.037**	0.055
		Benzaldehyde	0.408	**0.278**	0.474
		Nonan-2-one	0.063	**0.043**	0.063
		2-Phenylethanol	0.164	**0.112**	0.164
		Maltol	0.074	0.051	0.089
		Ethyl malto	0.130	**0.089**	0.130
		4-ethylguaiacol	0.497	**0.339**	0.496
		2-Phenylethyl acetate	1.297	**0.884**	1.295

		delta-Decalactone	0.136	**0.093**	0.136
		2-Acetylfuran	0.078	**0.053**	0.078
		Benzyl benzoate	0.109	**0.075**	0.109
		Ethyl tetradecanoate	0.063	**0.043**	0.063
		Ethyl tetradecanoate;Benzyl phenyl acetate	0.058	**0.040**	0.058
		Octyl acetate	0.104	**0.071**	0.104
**Human assesment**	**smell**	sour	1.873	2.082	3.055
		earthy	4.295	4.891	**7.055**
		maritime	3.868	3.227	**7.091**
	**taste**	bitter	6.514	4.109	**7.555**
		sour	2.895	2.991	**6.627**
		malty	6.295	6.236	**2.145**
		cereal	**6.500**	3.409	2.418

Explanatory notes:
SRS (sourness), GPS (metallic), STS (saltiness), SPS (spiciness), UMS (umami), SWS (sweetness), and BRS (bitterness)
